# Are Nurses Working in Mental Health in a Time of Changing Approaches More Exposed to Stress than other Professionals? Job Satisfaction and Perception of Respect for the Rights of Users in Seven Different Countries Worldwide

**DOI:** 10.2174/0117450179349117241211094816

**Published:** 2024-12-31

**Authors:** Cesar Ivan Aviles Gonzalez, Sara De Matteis, Yessika Madelaine Abarca Arias, Doris Marina Cerchiaro Fernandez, Martha Esther Guerra Munoz, Goce Kalcev, Maura Galletta, Maria Rita Pinna, Rober Romero Ramirez, Maria Veronica Brasesco, Uta Ouali, Fabrizio Bert, Mehmet Eskin, Massimo Tusconi, Mauro Giovanni Carta

**Affiliations:** 1 Department of Medical Sciences And Public Health, University of Cagliari, Cagliari, Italy; 2 Department of Health Sciences, University of Milan, Milan, Italy; 3 Department of Nursing, National University of Saint Augustine, Arequipa, Peru; 4 Department of Nursing, Popular University of Cesar, Valledupar, Colombia; 5 Faculty of Economic And Accounting Sciences, Popular University of Cesar, Valledupar, Colombia; 6 Department of Rectory, Popular University of Cesar, Valledupar, Colombia; 7 RIAT Red Internacional De Acompañamiento Terapéutico, Universidad Del Salvador, Buenos Aires, Argentina; 8 Psychiatry A, Razi Hospital, La Manouba, Manouba, Tunisia; 9 Department of Psychology, Koc University, Istanbul, Turkey; 10 Consultation Psychiatry And Psychosomatic Center, Azienda Ospedaliero-Universitaria di Cagliari, Cagliari, Italy; 11 Department of Public Health and Pediatric Sciences, University of Turin, 10126 Turin, Italy

**Keywords:** Human rights, Job satisfaction, Well-being, Mental health, Nursing, Mediterranean area, Multicenter study

## Abstract

**Background:**

The perception of respect for users' rights is fundamental for organizational well-being in mental health services. This cross-sectional observational study examined the job satisfaction and perception of user rights among nursing staff compared to other health professionals across seven countries in the Mediterranean and Latin American regions. This research measures this perception among nursing staff in different countries, with a particular focus on regional differences and professional roles.

**Methods:**

Data were collected from mental health services in four Mediterranean and three Latin American countries *via* a structured questionnaire (Well-Being at Work and Respect Rights Questionnaire - WWRR), administered both online and on paper. Using multivariable logistic regression, the study analyzed associations between job satisfaction, respect for rights, and various socio-demographic and professional factors.

**Results:**

Among 408 nurses and 492 other health professionals, findings revealed significant cross-country variability. Nurses in North Macedonia reported higher job satisfaction and perceived respect for user rights than other professionals (*p*<0.0001 in all items of the questionnaire), while Tunisian nurses showed lower organizational satisfaction but higher respect for user rights (*p*=0.033 for respect for rights). However, in general, no significant differences were found between nurses and other professionals across all items.

**Conclusion:**

The perception of respect for users' rights is fundamental for organizational well-being in mental health services. The study highlights differences in organizational well-being perceptions across various contexts, emphasizing the need for culturally and economically adapted policies to improve mental health service environments globally. The results indicate the variability in the perception of respect for rights and job satisfaction between different geographical and professional contexts. This indicates the need for policies adapted to specific cultural and economic realities to improve organizational well-being in mental health services.

## 1 INTRODUCTION

The policies that promote psychiatric care from the centrality of hospital care to community care involve modifications of the roles of health professionals [1-3]. Among all the figures, among those having a higher impact by the transformation policies are nurses, whose role is linked to consolidated skills in a hospital care setting, but they find uncertain boundaries and, in many respects, to be defined in a community care system [4]. All this can increase the risk of burnout or just job dissatisfaction even in situations in which the transformation is started to be barely mentioned in practice, but the principles that inspire it are novelty shared [5]. Another element that accentuated the working risk conditions for nurses was the COVID-19 pandemic.

In countries in which the change in the mental health system seems more consolidated, such as Italy, a country considered at the forefront in the implementation of community assistance policies in mental health, the situation of the nurse in the context of community assistance was no longer at risk than other professionals [6, 7]. However, even in this country and in other countries with developed community care networks, some recent phenomena have changed the situation of the nurse in the mental health system. Firstly, the economic crisis has reduced resources for community care and, secondly, the pandemic crisis has highlighted a greater risk of stress in nurses compared to other health professionals, perhaps because they are more exposed to risk [8-10].

A recent line of research by our group has supported the hypothesis that the perception of respect for the rights of people with psychosocial disabilities by health workers in mental health centers is an essential element of the organizational well-being of these structures, *i.e*., the greater the perception that user rights are respected, the greater the well-being and satisfaction of workers [11-13].

Given our premises on the specific role of the nurse in mental health services in this historical moment of transition (and after the strong stressing COVID era), it may be interesting to investigate whether there is a specificity of this figure in the perception of organizational well-being and the related perception of the user's rights.

Our work was inspired by the United Nations Convention on the Rights of Persons with Disabilities (CRPD) which highlighted the relevance of the respect of rights and the active participation of people with disabilities in decisions affecting their lives, including around health treatments [14]. This issue is especially critical in the context of psychosocial disabilities, where discrimination and human rights violations are frequent [15]. In fact, although mental health conditions account for one-third of the global burden of disability [16], more than 70% of those having needs do not have access to quality services [17]. Prior to the Covid-19 pandemic, spending on mental health was less than 2 dollars per person per year and even less in low-income countries [18]. However, this expenditure has further decreased due to the pandemic [19].

In response to these challenges, the World Health Organization's QualityRights program seeks to incorporate the principles of the CRPD in the field of psychosocial disability [20, 21].

The condition of the nurse in mental health care is, therefore, that of a critical professional figure in a critical care area. Our research primarily aims to evaluate how the organizational well-being of nurses in mental health services in the selected countries affects and is affected by the perception of respect for their rights and those of the users. Furthermore, it seeks to describe the differences and similarities in the experiences of nurses in these countries. Through a descriptive quantitative approach, these dynamics will be explored, allowing a detailed and comparative analysis of the data collected in different settings and facilitating the identification of key patterns and differences between the studied regions.

## 2 METHODS

### 2.1 Design

The Design of the study was cross-sectional and observational. Data collection began in October 2018 and concluded in July 2019. The study was conducted across mental health services of four Mediterranean countries (Gaza, Italy, North Macedonia, and Tunisia) and in three of Latin America (Argentina, Colombia, and Peru).

### 2.2 Settings and Sampling

A convenience sampling method was adopted in Latin American Countries for health professionals working in private and public mental health services. In the four Mediterranean Countries, a random sample (1/3) representative of staff of the mental health care network was interviewed. The survey questionnaires were administered both online and using face-to-face paper-based methods. All the health workers of mental health services take part in the study (nurses, psychiatrists, rehabilitators, and psychologists, administrative staff).

- In Argentina, the study involved health professionals both of community mental health services and hospital departments of the metropolis of La Plata,
- In Colombia, the research was implemented in one public and private mental health service in Valledupar, Caribbean region. Mental health professionals from clinics, ambulatory care centers, and public health institutions were involved.
- In Peru, professionals working both in psychiatric hospital units and mental health centers were involved across the country.
- In Tunisia, the research was implemented at Razi Hospital, the only Tunisian psychiatric hospital in the southwestern Tunis metropolitan area. The hospital consists in seven adult psychiatric inpatient wards, one forensic psychiatry ward, one adult outpatient unit, and one child and adolescent psychiatry ward.
- In North Macedonia, the survey was conducted on health professionals in private services for Psychotherapy and Counseling, Mental Health, and Psychology.
- In South Sardinia (Italy), the study was carried out in the “Department of Mental Health ASL8”. This is a community public health care network supporting an area of 500,000 adult inhabitants in the southwest of Sardinia, including the city of Cagliari. It includes several community care services and two psychiatric hospital wards.

### 2.3 Instruments

Each health worker participant completed a sheet for socio-demographic personal data and fulfilled the Well-Being at Work and Respect Right Questionnaire (WWRR); it is a seven-item questionnaire. The first five items adopted a Likert scale for coding where 1 was “Not satisfied at all” and 6 was “Totally satisfied”. The questions were:

(1) How much are you satisfied with your job?

(2) How much do you think the users of your service ward are satisfied?

(3) How much are you satisfied with the organizational aspect of your work/how your work is organized?

(4) How much do you think the human rights of the users of your service/ward are respected?

(5) How much do you think the human rights of your staff are respected?

In item 6, the coding runs in the opposite direction (1 was “Totally satisfied”).

(1) How do you evaluate the current state of care in mental health in your service/ward, with reference to resources?

The seventh item uses a nominal scale choice and asks about perceptions regarding the need for specific human resources for the mental health service.

### 2.4 Definitions of Outcomes and Key Variables

The primary outcome of this study is job satisfaction among mental health nurses, measured using a seven-item Well-Being at Work and Respect Rights Questionnaire (WWRR). Additional outcomes include perceived respect for user rights and organizational satisfaction, as rated by participants on a Likert scale. The main exposure is the professional role in mental health services (*i.e*., whether the participant is a nurse or another type of health professional). This categorization allows a comparison of perceptions and satisfaction levels between nurses and other healthcare providers. Key predictors include sociodemographic factors (*e.g*., age, gender), professional characteristics (*e.g*., years of experience, type of health facility), and regional location (*i.e*., country), as these may influence job satisfaction and perceptions of rights and respect. Potential confounders include age, years of professional experience, type of health service (*e.g*., hospital-based *vs*. community-based), and geographical region. These factors were considered as they may impact job satisfaction independently of the main exposures and outcomes. The study also considers country-specific economic and cultural contexts as possible effect modifiers, recognizing that varying conditions in the Mediterranean and Latin American regions could modify the relationships between job satisfaction, respect for user rights, and professional roles. No formal diagnostic criteria were applied in this study, as it focused on perceptions and self-reported measures rather than clinical diagnoses. To mitigate sampling bias, we used a combination of random sampling in the Mediterranean countries and convenience sampling in the Latin American countries. This approach aimed to balance practicality with representativeness. The random sampling method in the Mediterranean countries (selecting approximately one-third of the mental health workforce) improves generalizability within these regions.

### 2.5 Statistical Analysis

Continuous variables were presented as means with standard deviations, while categorical variables were described using counts and percentages. This approach provides a clear overview of the data’s central tendency and distribution.

The association between the answers to the WWRR questionnaire by nurses and other professionals (dependent variable) and biographic, working role and geographical data (explanatory variables) was preli- minarily examined in a univariate analysis using the chi-squared test quantifying the association by unadjusted variance. Subsequently, a multivariable analysis was performed using binary logistic regression, adjusting for confounding factors and assessing effect modification if needed. All analyses were performed on both the complete-case dataset and the imputed dataset. Results were compared to ensure consistency, and any substantial differences were reported in the results section to provide transparency on the impact of missing data. Quantitative variables, such as age and years of professional experience, were initially analyzed as continuous variables. Descriptive statistics (means and standard deviations) were calculated to summarize the central tendencies and distributions of these variables across the sample. Continuous variables were examined in their original form to retain precision and maximize the power of the analysis. The model construction was done using backward-selection procedures. Associations between the answers to the questionnaire and the independent variables were presented as Z test was calculated by Adjusted variance. A p-value < 0.05 was considered statistically significant.

All analyses were performed using Stata statistical software (version 17.0, Stata Corporation, College Station, Texas, USA).

### 2.6 Ethics

The Institutional Review Board of the University Hospital of Cagliari, Italy, approved the protocol of the study, which has been conducted in accordance with the guidelines of the 1995 Declaration of Helsinki and its revisions (protocol number PG/2018/7337).

## 3 RESULTS

Table **1** illustrates the characteristics of the sample of nurses recruited for the study, and the Italian sample is clearly more represented in the last two categories by age, *i.e*., those over 49 years of age (75% in Italy *versus* 19.9% in the rest of the countries, chi-square 161, 445, *p*<0.0001); while in Argentina women are more frequent (85% *vs* 60.2% in the rest of the nations, chi-square 26, 442, *p*<0.0001). The primary exposure was the professional role, with comparisons made between nurses and other health professionals regarding job satisfaction, organizational well-being, and respect for users' rights. Geographic location (Mediterranean *vs*. Latin American countries) was also considered, as it potentially modifies the effects of professional role on these outcomes.

The primary objective of this study was to evaluate the differences in job satisfaction, organizational well-being, and perception of respect for user rights among mental health nurses compared to other health professionals across diverse geographic contexts.

Table **2** shows the comparison between nurses and other health professionals in the responses to the questionnaire. In none of the responses was a statistically significant difference in answers between nurses and other health professionals, even if on item 3 (How much are you satisfied with the organizational aspect of your work/how is your work organized?) the difference, in the sense of lower satisfaction in nurses, is at the limit of statistical significance (3.73±1.43 in nurses *versus* 3.91±1.42 in other professionals, Z=1.852, *p*=0.0641). However, when comparing between individual countries (Table **3**), divergent scores at responses with higher score among nurses in Macedonia in items 1 “How satisfied are you with your job?” (chi square, 5 df, = 41.391, *p*<0.0001); Item 2 “How satisfied do you think the users of your service department are?” (chi square, 5 df, = 33.216, *p*<0.0001); Item 3 “How satisfied are you with the organizational aspect of your work/how your work is organised?” (chi square, 5 df, = 42.604, *p*<0.0001); Item 4 “How much do you think the human rights of the users of your service/department are respected?” (chi square, 5 df, = 35.903, *p*<0.0001); Item 5 “How much do you believe that the human rights of your staff are respected?” (chi square, 5 df, = 48.8228, *p*<0.0001), and lower in Item 6 (in which, however, the value is reversed, *i.e*. lower scores correspond to better satisfaction) “How do you rate the current state of mental health care in your service/department, with reference to resources?” (chi square, 5 df, = 36.771, *p*<0.0001); in Tunisia, in reverse, some items showed low scores in nurses: Item 1 “How satisfied are you with your job?” (chi square, 5 df, = 14.753, *p*=0.011); Item 3 - “How satisfied are you with the organizational aspect of your job/how your job is organized?” (chi square, 5 df, = 11.959, *p*=0.035), but with Item 4 showing a higher score in nurses “How much do you think the human rights of the users of your service/department are respected?” (chi square, 5 df, = 12.141, *p*=0.033).

## 4 DISCUSSION

Our study has highlighted, in partial contrast with the initial hypothesis, that, considering all the countries analyzed, no differences emerge between the responses of nurses and those of other healthcare professionals working in mental health on job satisfaction, perception that users are satisfied by the care they receive, satisfaction with the organizational aspects at work, perception that the human rights of the users of your service/ward are respected, perception that the human rights of the staff where you work are respected, satisfaction with the current state of care in mental health in your service/ward, with reference to availability resources?

In this work, the comparisons between nations were made by comparing the differences between nurses and other professionals within the nations and not as absolute values of the nurses' responses in each specific nation, on the assumption that these values in nurses alone are extremely influenced by contingent variables because a direct comparison is reasonably discussed, while a specific national difference between nurses and other professionals would have been more useful for verifying the starting hypotheses. From this point of view, when we evaluate what happens in individual nations, we find that in North Macedonia, nurses respond with scores that show greater satisfaction than other professionals in all the items considered, while in Tunisia, nurses express lower scores in the items relating to job satisfaction, satisfaction about organization at work but higher considering respect for user rights.

How to explain these results?

First, both North Macedonia and Tunisia have had to face serious emergencies in the organization of health services and mental health services. In North Macedonia, tubular spending on health has declined in recent years, it is among the lowest in Southeast Europe [22] and “There is strong reliance on out-of-pocket payments which represent 40.4% of total health spending, one of the highest shares in South-Eastern Europe” [22]. The general health emergency has affected mental health to an even greater extent, which has suffered mainly the impact of the pandemic [23]. This is why the renewal plans have tried to involve this area, too. After fierce protests from healthcare workers in the years preceding 2022 (in which these data were collected), the government promoted a general reform involving general practitioners in the provision of specialist care. Following the collection of our data, the first corrective interventions were conducted by the government with the support of the WHO. Some recent reports have noted several “remaining challenges” [24], such as insufficient number of health professionals, low number of medications covered by health insurance, transportation barriers for accessing health services in rural areas, inadequate gender norms and waiting time in obtaining appointments [24]. In this context we cannot say that the nurses were satisfied, but the most dissatisfied of all were the doctors who, also on an economic level, had salaries that were proportionately lower than in neighboring European countries.

Tunisia's health situation appears equally critical. The country is experiencing a serious economic crisis that has been worsened by drought and impact of COVID-19 pandemic [25]. It has drastic repercussions on the salaries of staff employed in the public healthcare system and on the availability of drugs and healthcare products [26]. The fact that there are well-structured public universities plays a paradoxically negative role because well-trained, multilingual doctors, psychologists, and nurses find it easy to emigrate, especially to the rich Gulf countries and French-speaking countries such as Canada, Belgium, France and Switzerland. The result is a drastic shortage of personnel, which has a greater impact on the most often penalized healthcare areas, such as mental health [20, 26]. The fact that nurses present higher levels of job dissatisfaction compared to other health professionals in a context in which everyone is dissatisfied with their salaries, and nurses are the most dissatisfied of all because, in terms of the economic unsustainability of daily life, they are the ones who have the lowest net salaries (around 400 Euros / Month) compared to doctors, psychologists, and administrative staff members. The discrepancy, already highlighted in Tunisia concerning all professionals, in the correlation between job satisfaction and perception of respect for users' rights should be interpreted in the light of the above mentioned situation [11]. Indeed, in this context, the perception of non-respect for one's rights and the consequent anger could lead to not paying attention to the rights of others [11].

While not fully confirming the starting hypotheses, however, some of the starting hypotheses are confirmed by our results, that is, due to their position of close contact with users, strong proximity to them, and the strong impact of the profession in terms of potential stress, nurses are the most fragile segment of the healthcare system in the event of a crisis. In the case of North Macedonia and Tunisia, however, the pandemic may have been a co-factor of the critical situation but not the only element, while the crisis of the transformation of care towards a community-centered system does not seem to have played a relevant role. The real crisis seems to be caused in the two nations by strictly economic issues.

Another relevant element is that in two nations with a critical situation of mental health care, which causes greater dissatisfaction in overall professionals, nurses respond in the opposite way in relation to the specific context, just as the perception of users' rights is opposite. Specific elements linked to culture and specific working conditions probably produce the differences while interacting with general and universally applicable values linked to concepts relating to human rights.

In general, the already demonstrated relationship between job satisfaction and perception of respect for rights [27, 28] is confirmed, although there are some cases of extreme job dissatisfaction, as in Tunisia, in which we found opposite results.

Our study offers valuable insights into the experiences and perceptions of mental health professionals, but several limitations should be acknowledged. The self-report nature of the questionnaire could introduce response biases, as participants may respond in socially acceptable ways. The voluntary participation method may have led to a non-representative sample, skewing results due to motivated individuals being more likely to participate. Furthermore, although the sample size of 900 participants appears adequate for the study, ensuring good representativeness of national groups and backgrounds, however, due to the very multicenter nature of the study, differences in sampling methods between countries could affect the comparability of the data and should be taken into account when analysing. This fact should be considered equally with the possibility of evaluating data from clinical settings that appear in some cases to be difficult to reach and investigate.

The response rate could not be determined, affecting the representativeness of the data. Additionally, the dual administration modes (online and paper) might have influenced responses, impacting data consistency. Moreover, country-specific factors and the predominance of nurses and psychologists in the sample could further affect the findings.

## CONCLUSION

The study seems to confirm that nurses represent the category most sensitive to critical situations that can impact the quality of care provided and this influences both job satisfaction and the perception of respect for user’s rights. However, given the situation in the nations examined, the most critical conditions highlighted concern general economic factors and not, as expected, the transition from hospital care to community care.

## Figures and Tables

**Table 1:** General characteristics of the participants.

**-**	**Italy**	**North Macedonia**	**Palestine**	**Tunisia**	**Argentina**	**Colombia**	**Peru**
**-**	**N = 126**	**N = 100**	**N = 164**	**N = 200**	**N = 110**	**N= 101**	**N = 99**
Men Women	45 (36%) 81 (64%)	39 (39%) 61 (61%)	88 (54%) 76 (46%)	78 (39%) 122 (61%)	16 (14.5%) 94 (85.5%)	34 (33.7%) 67 (66.3%)	40 (40.4%) 59 (59.6%)
<20 years old 20-29 years ì30-39 years ì40-49 years ì50-59 years >60 years	0 (0%) 0 (0%) 2 (2%) 30 (24%) 74 (59%) 20 (16%)	4 (4%) 21 (21%) 19 (19%) 28 (28%) 22 (22%) 6 (6%)	0 (0%) 28 (17% 53 (32%) 46 (28%) 34 (21%) 3 (2%)	1 (0.5%) 60 (30%) 79 (39.5%) 30 (15%) 29 (14.5%) 1 (0.5%)	0 (0.00%) 34 (30.9%) 36 (32.7%) 20 (18.2%) 18 (16.4%) 2 (1.8%)	0 (0.00%) 20 (19.8%) 42 (41.6%) 16 (15.8%) 12 (11.9%) 11 (10.9%)	1 (1.00%) 16 (16.2%) 42 (42.4%) 24 (24.2%) 11 (11.1%) 5 (5.1%)
Nurses	74	40	69	118	4	62	41

**Table 2:** Comparison between nurses and other health professionals.

-	Nurses N=408 Mean (SD)	Others N=492 Mean (SD)	Z	P
Item 1 - How much are you satisfied with your job?	4.10 (1.40)	4.08 (1.38)	-0.202	0.8397
Item 2 - How much do you think the users of your service ward are satisfied?	4.23 (1.40)	4.25 (1.40)	0.115	0.9081
Item 3 - How much are you satisfied with the organizational aspect of your work/how your work is organized?	3.73 (1.43)	3.91 (1.42)	1.852	0.0641
Item 4 - How much do you think the human rights of the users of your service/ward are respected?	4.38 (1.49)	4.29 (1.47)	-0.978	0.3282
Item 5 How much do you think the human rights of your staff are respected?	3.81 (1.69)	3.88 (1.62)	0.399	0.6902
Item 6 How do you evaluate the current state of care in mental health in your service/ward, with reference to resources?	3.48 (1.04)	3.44 (1.13)	-0.682	0.4950

**Table 3:** Comparison between nurses and other health professionals by countries.

-	Argentina (n=110) Mean (SD)	Colombia (n=101) Mean (SD)	Peru (n=99) Mean (SD)	Palestine (n=164) Mean (SD)	North Macedonia (n=100) Mean (SD)	Tunisia (n=200) Mean (SD)	Italy (n=126) Mean (SD)	Total (n=900) Mean (SD)
Item 1 - How much are you satisfied with your job? Nurses	4.00 (1.41)	4.35 (1.28)	4.66 (1.11)	3.86 (1.33)	4.28 (1.01)	3.87 (1.73)	4.09 (1.22)	4.10 (1.40)
Item 1 - Others	4.50 (1.41)	4.67 (0.96)	4.97 (0.62)	3.64 (1.35)	2.81 (1.29)	4.00 (1.36)	4.21 (1.07)	4.08 (1.38)
chi^2^(5 df)	3.6127	5.0530	6.6650	2.3436	41.3918	14.7530	3.7906	4.7203
p	0.606	0.409	0.247	0.800	<0.0001	0.011	0.580	0.451
Item 2 - How much do you think the users of your service ward are satisfied? Nurses	4.75 (1.50)	4.55 (1.12)	4.97 (0.69)	4.00 (1.26)	4.70 (0.79)	3.79 (1.68)	4.19 (1.04)	4.23 (1.32)
Item 2 - Others	5.00 (1.15)	4.87 (0.98)	4.84 (0.59)	3.83 (1.28)	3.38 (1.25)	3.57 (1.42)	4.38 (0.91)	4.25 (1.31)
chi^2^(5 df)	2.9401	3.1687	2.6279	3.6470	33.2161	8.4693	3.2687	0.7153
p	0.709	0.674	0.453	0.601	<0.0001	0.1321	0.514	0.982
Item 3 - How much are you satisfied with the organizational aspect of your work/how your work is organized? Nurses	5.00 (0.82)	3.76 (1.39)	4.71 (0.87)	3.86 (1.12)	4.50 (0.91)	3.09 (1.72)	3.58 (1.16)	3.73 (1.43)
Item 3 - Others	4.65 (1.44)	4.56 (0.88)	4.72 (0.69)	3.66 (1.34)	2.98 (1.28)	3.14 (1.36)	3.71 (1.24)	3.91 (1.42)
chi^2^(5 df)	2.1334	12.0573	2.3308	6.2090	42.6046	11.9592	3.2312	10.9235
p	0.830	0.034	0.675	0.286	<0.0001	0.035	0.664	0.053
Item 4 - How much do you think the human rights of the users of your service/ward are respected? Nurses	4.75 (1.26)	4.71 (1.29)	4.66 (0.99)	3.65 (1.36)	5.08 (0.73)	3.97 1.82()	4.85 (1.22)	4.38 (1.48)
Item 4 - Others	4.89 (1.27)	5.23 (0.87)	4.79 (0.77)	3.59 (1.55)	3.68 (1.38)	3.54 (1.56)	5.02 (1.06)	4.29 (1.46)
chi^2^(5 df)	3.2213	6.0151	1.8832	6.2725	35.9037	12.1412	1.7863	4.6960
p	0.666	0.305	0.865	0.281	<0.0001	0.033	0.878	0.454
Item 5 How much do you think the human rights of your staff are respected? Nurses	3.75 (2.22)	4.81 (1.27)	4.43 (1.29)	3.67 (1.47)	5.23 (0.53)	2.51 (1.76)	4.05 (1.23)	3.81 (1.69)
Item 5 - Others	4.37 (1.57)	5.21 (0.98)	3.02 (1.48)	3.56 (1.46)	3.63 (1.44)	2.31 (1.48)	4.14 (1.29)	3.88 (1.62)
chi^2^(5 df)	2.7094	4.6469	9.5684	3.9030	48.8228	5.9135	0.9820	10.2136
p	0.745	0.460	0.088	0.563	<0.0001	0.315	0.964	0.069
Item 6 How do you evaluate the current state of care in mental health in your service/ward, with reference to resources? Nurses	3.25 (1.25)	3.58 (0.97)	3.34 (0.79)	3.22 (1.29)	3.28 (0.96)	3.87 (0.97)	3.23 (0.91)	3.48 (1.04)
Item 6 - Others	2.92 (0.95)	4.00 (1.21)	1.97 (0.18)	3.00 (1.27)	4.40 (0.87)	3.88 (0.96)	3.50 (0.64)	3.44 (1.13)
chi^2^(5 df)	6.5626	6.9786	3.6613	2.6381	36.7714	1.3697	7.4471	8.3577
p	0.161	0.222	0.454	0.620	<0.0001	0.849	0.114	0.138

## Data Availability

The data and supportive information are available within the article.

## LIST OF ABBREVIATIONS

CRPD: = Convention Rights Persons Disabilities
WWRR: = Work Respect Right Questionnaire

## STANDARDS OF REPORTING

STROBE guidelines were followed.
